# Targeted Therapies for Multiple Myeloma

**DOI:** 10.3390/jpm11050334

**Published:** 2021-04-23

**Authors:** Christopher Chang-Yew Leow, Michael Sze Yuan Low

**Affiliations:** Monash Haematology, 246 Clayton Road, Clayton, VIC 3168, Australia; chris.leow@monashhealth.org

**Keywords:** multiple myeloma, therapeutic targets, monoclonal antibody

## Abstract

Multiple myeloma continues to be a challenging disorder to treat despite improved therapies and the widespread use of proteasome inhibitors and immunomodulatory drugs. Although patient outcomes have improved, the disease continues to invariably relapse, and in the majority of cases, a cure remains elusive. In the last decade, there has been an explosion of novel drugs targeting cellular proteins essential for malignant plasma cell proliferation and survival. In this review, we focus on novel druggable targets leading to the development of monoclonal antibodies and cellular therapies against surface antigens (CD38, CD47, CD138, BCMA, SLAMF7, GPRC5D, FcRH5), inhibitors of epigenetic regulators such as histone deacetylase (HDAC), and agents targeting anti-apoptotic (BCL-2), ribosomal (eEF1A2) and nuclear export (XPO1) proteins.

## 1. Basic Biology of Plasma Cell Neoplasms

Multiple myeloma (MM) is a neoplastic disorder characterised by the abnormal proliferation of antibody-secreting plasma cells (PCs). The malignancy is part of a heterogeneous spectrum of disorders ranging from the indolent pre-cursor condition, monoclonal gammopathy of undetermined significance (MGUS), to the highly aggressive plasma cell leukaemia [1]. Whilst early stages of the disease are asymptomatic, progression is heralded by end-organ symptoms, including hypercalcaemia, renal failure, anaemia and lytic lesions.

Symptomatic MM is thought to evolve from the expansion of altered clones that are already present within the precursor MGUS. Although these initial events may generate a malignant clone, a “second” hit such as dysregulation of cell cycle control, evasion of normal apoptotic pathways or stimulation by the abnormal bone marrow (BM) microenvironment are required for myelomagenesis [2,3]. Karyotypic abnormalities account for the majority, if not all, of the initiating events, with a significant proportion identified in MGUS patients [4]. A dichotomy of genetic aberrations is observed. The first group is hyperdiploid (near double the number of normal chromosomes) with a specific genotype involving aneuploidy of several odd-numbered chromosomes, including 3, 5, 7, 9, 11 and 21. The second group involves translocations of the IgH locus at chromosome 14q. These translocations juxtapose the IgH promoter to one of many oncogenes, including three Cyclin D genes (*CCND1-3*), *WHSC1* (*MMSET*), *MAF* or *MAFB* [5].

Progression of MGUS to MM is regularly accompanied by secondary genetic events such as *Myc* translocations (chromosome 8q42), leading to increased cell cycling and proliferation, the loss of cell cycle arrest signals including *p53* (deletion 17p) and *CDKN2C* (deletion 1p), and genetic aberrations with less understood consequences such as deletion 13q and amplification 1q [5,6]. Additionally, aberrant NFκB and Ras signalling are a relatively common event in MM and arise from a broad mutation spectrum that provides a myriad of pro-survival and growth signals to MM cells [7,8,9]. Although these genetic alterations appear to be the drivers of tumorigenesis, they manifest in different phenotypes under the influence of epigenetic mechanisms. Such epigenetic changes involve DNA methylation and primarily occur on CpG dinucleotides [10]. Increasing evidence demonstrates that dysregulation of epigenetic regulators such as EZH2, DNMT3A and histone deacetylases (HDACs) promote and contribute to the complexity of myelomagenesis, whilst also providing potential therapeutic targets [11,12,13].

Lastly, interactions between malignant PCs and the bone marrow (BM) milieu promote malignant transformation and propagation. IL-6 has been demonstrated to be an essential promoter of malignant PC survival and an inhibitor of apoptosis via numerous pathways, such as upregulation of the pro-survival proteins, BCL-xL and MCL-1 [14,15]. Dysregulation of other cytokines in the BM microenvironment results in angiogenesis (VEGF), evasion of cell-mediated immunity (TNF-alpha and IL-12) and escape from pro-apoptotic pathways (PDGF, IGF-1) [16]. These cytokines and soluble factors all provide potential targets for drug therapy.

## 2. Standard-of-Care Therapy for Multiple Myeloma

With expanding knowledge of PC and myeloma biology, treatment of MM has become more effective. The advent of proteasome inhibitors (PIs) and immunomodulatory drugs (IMiDs) has markedly improved the response rates and significantly increased survival in MM patients [17]. PIs (such as bortezomib) prevent the degradation of misfolded proteins via the proteasome, resulting in their accumulation within a malignant PC and eventual cell death due to the unfolded protein response [18]. The proteasome is also involved, with additional cellular functions, including the regulation of signalling pathways, cell cycle and DNA repair [19], hence, PIs may promote MM cell death through a variety of mechanisms. IMiDs (such as thalidomide and lenalidomide) function by altering the target specificity of CUL4A-DDB1-Cereblon E3 ubiquitin ligase, which in MM, leads to the degradation of two key PC transcription factors, Ikaros (IKZF1) and Aiolos (IKZF3) [20,21]. IMiD-mediated degradation of IKZF1 and IKZF3 results in a reduction in IRF4, a key mediator of the oncogene MYC [22]. Furthermore, IKZF1 and IKZF3 degradation results in MM cell growth arrest and the activation of T-cells, both of which contribute to the anti-myeloma effect of IMiDs [20,21].

Combinations of these agents form the pillars of frontline treatment of MM. Three drug regimens utilising bortezomib, dexamethasone and an additional agent, such as cyclophosphamide (VCd), thalidomide (VTd) or lenalidomide (RVd), are the standard of care prior to autologous stem cell transplantation (ASCT), which utilises a melphalan-based conditioning regimen [23]. Following ASCT, bortezomib- or lenalidomide-based maintenance therapy is commenced to prolong response, slow progression and improve overall survival [24,25,26]. Patients ineligible for an ASCT are still evaluated for induction with bortezomib, lenalidomide or an alkylating-agent based regimen. Newer PIs (carfilzomib, ixazomib) and IMiDs (pomalidomide) have demonstrated improved progression-free survival (PFS) and overall survival (OS) in an assortment of regimens in relapsed/refractory MM (RRMM) patients [27,28,29,30]. Further novel PIs (maprozomib, oprozomib) are also currently being investigated [31]. However, in the absence of a cure, malignant PC clones evolve to become increasingly aggressive and refractory to PI and IMiD therapies, culminating in disease relapse, progression and death.

## 3. Novel Therapies

### 3.1. Monoclonal Antibodies Targeting Cell Surface Antigens

#### 3.1.1. CD38

CD38 is a type II transmembrane glycoprotein whose ascribed functions include receptor-mediated adhesion, signal transduction in lymphoid and myeloid cells and bifunctional ecto-enzymatic activities that modulate intracellular calcium mobilization [32]. CD38 is expressed at minimal levels on normal lymphoid and myeloid cells and in some non-haematopoietic tissues as well as red blood cells [33]. The high expression of CD38 on malignant plasma cells in MM and its role in cell signalling have made it an attractive antibody target [34].

Daratumumab is a human IgG1κ antibody directed against a unique CD38 epitope [35]. The anti-myeloma activity of daratumumab is mediated by a diverse range of mechanisms including antibody-dependent cellular cytotoxicity (ADCC) and phagocytosis, complement-dependent cytotoxicity (CDC), apoptosis and inhibition of the enzymatic effects of CD38 [36]. Rapid FDA approval of daratumumab occurred in 2016 following the results of two phase I/II clinical trials using single-agent daratumumab in RRMM [37,38]. The final analysis of patients enrolled in these studies demonstrated an overall response rate (ORR) of 31.1%, a median duration of response (DOR) of 7.6 months and a median PFS and OS of 4.0 months and 20.1 months, respectively [39]. Subsequently, daratumumab has been investigated in conjunction with established combinations of anti-myeloma therapies.

The addition of daratumumab to PIs was investigated in the phase III CASTOR trial which analysed the effect of the addition of daratumumab to bortezomib and dexamethasone (DVd versus Vd) in 498 RRMM patients. The ORR was higher in the daratumumab group (82.9% versus 63.2%, *p* = 0.001) [40]. Longer term follow-up revealed that the addition of daratumumab led to improved progression-free survival (16.7 vs. 7.1 months; *p* < 0.0001). Infusion-related reactions associated with daratumumab treatment were common (45.3%), predominantly grade 1/2 (91.4%), and the majority occurred during the first infusion (98.2%). Subgroup analysis of the CASTOR trial based on cytogenetic risk has demonstrated that DVd prolonged the median PFS in patients with standard (16.6 vs. 6.6 months; *p* < 0.0001) and high (12.6 vs. 6.2 months; *p* = 0.00106) cytogenetic risks. Overall, DVd achieved deeper responses with higher rates of MRD negativity and sustained MRD negativity regardless of cytogenetic risk [41]. The addition of daratumumab to carfilzomib (CANDOR and EQUULEUS trial) has also been demonstrated to improve PFS [42,43].

Similar studies combining daratumumub and IMiDs have shown equally impressive results with the phase III POLLUX study comparing the addition of daratumumab to lenalidomide and dexamethasone (DRd versus Rd) in 569 patients with RRMM [44]. After 44.3 months median follow-up, DRd versus Rd demonstrated higher ORR (92.9% vs. 76.4%; *p* < 0.0001) and median PFS (44.5 vs. 17.5 months; *p* < 0.0001). Deeper responses, including CR or better (56.6% vs. 23.2%; *p* < 0.001) and MRD negativity (10^−5^; 30.4% vs. 5.3%; *p* < 0.0001) were observed. The addition of daratumumab to other IMiDs has been evaluated in the phase II MM-014 trial, which showed an impressive ORR of 77.7% with daratumumab plus pomalidomide and dexamethasone (Pd), with all patients having received lenalidomide in their most recent prior regimen (75% lenalidomide refractory) [45]. Recent data from the APOLLO study utilizing subcutaneous daratumumab in conjunction with Pd have demonstrated a similar benefit, with a reduction in risk of progression or death by 37% in RRMM [46]. Evidence suggests that treatment of MM with IMiDs leads to a transcriptional upregulation of CD38 resulting in increased CD38 cell surface expression, allowing for increased ADCC and NK cell killing with daratumumab [47]. This transcriptional change makes the combination of IMiDs and CD38 antibodies a particularly attractive therapy for MM patients.

Given its success in RRMM, the addition of daratumumab has recently been evaluated as part of upfront therapy for newly diagnosed myeloma. The ALCYONE trial compared the addition of daratumumab to bortezomib, melphalan and dexamethasone (D-VMP vs. VMP) in 706 newly diagnosed MM patients not eligible for autologous bone marrow transplantation [48]. The addition of daratumumab demonstrated an improved ORR (90.9% vs. 73.9%; *p* < 0.0001). Additionally, at median follow-up of 40.1 months D-VMP showed improved PFS and OS (36-month estimate: 78.0% vs. 67.9%; *p* = 0.0003) when compared to VMP alone. Undertaken in a different patient group, the CASSIOPEIA trial compared the addition of daratumumab to bortezomib, thalidomide and dexamethasone (D-VTD vs. VTD) as pre-transplant induction and as post-transplant consolidation in 1085 transplant-eligible newly diagnosed myeloma patients [49]. Whilst the addition of daratumumab led to no statistically significant change in ORR, a higher number of patients achieving a complete response or better (39% vs. 26%; *p* < 0.0001) and an improved PFS (HR 0.47; 95% CI 0.33–0.67; *p* < 0.0001) was observed. Currently, no OS benefit has been reported from the CASSIOPEIA trial.

At least three other CD38 monoclonal antibodies are under investigation for MM treatment. Isatuximab (SAR650984) is a chimeric IgG1 CD38 monoclonal antibody targeting a different epitope of CD38 than daratumumab [50]. Single-agent isatuximab demonstrated a reasonable ORR of 40.9%, with median PFS and OS of 4.6 and 18.7 months, respectively [51]. Isatuximab in combination with lenalidomide and dexamethasone demonstrated and ORR of 56% (29/52) [52]. The phase III ICARIA-MM study evaluated isatuximab, pomalidomide and dexamethasone versus Pd alone in 307 RRMM patients [53]. At a median follow-up of 11.6 months, median PFS was greater in the isatuximab, pomalidomide and dexamethasone arm (11.5 vs. 6.4 months; HR 0.596, 95% CI 0.44–0.81; *p* = 0.001). In March 2020, the FDA approved isatuximab (SARCLISA) in combination with Pd for adult patients with multiple myeloma who have received at least two prior therapies, including lenalidomide and a proteasome inhibitor. Isatuximab is currently being evaluated in combination with Vd, Kd, Rd and RVd in newly diagnosed and RRMM patients in a number of clinical trials [54,55,56]. Additional anti-CD38 monoclonal antibodies (MOR202, TAK-079) have been examined in pre-clinical studies [57]. Phase 1/2 data exploring MOR202 and TAK-079 have recently been published, and further clinical evaluation is underway [58,59]. Overall, CD38 monoclonal antibodies have demonstrated reasonable but not spectacular single-agent efficacy in RRMM patients; however, their use in combination with established therapies appears to significantly improve response rates and survival. In newly diagnosed MM patients, CD38 monoclonal antibodies appear effective and, in the right combination, improve survival, particularly in those who are ineligible for upfront autologous bone marrow transplantation.

#### 3.1.2. CD138

CD138 (syndecan-1) is a transmembrane protein receptor that is expressed on malignant PCs as well as epithelial cells and is used as primary diagnostic marker for MM [34,60]. Indatuximab ravtansine (BT062) is an antibody–drug conjugate (ADC) consisting of the anti-chimerised monoclonal antibody (nBT062) and the microtubule binding agent maytansinoid DM4 toxin. Indatuximab ravtansine specifically binds to CD138-expressing cells and, once internalised, DM4 is released to cause cell cycle arrest via tubulin binding followed by apoptosis [61,62]. Phase I/II data of the ADC as a single-dose or multi-dose regimen in RRMM demonstrated a disappointing ORR for this drug as a single agent of 6% and a short median time to progression (1.8 months) [63]. Similar to CD38 monoclonal antibodies, limited single-agent efficacy has led to indatuximab ravtansine being investigated in combination with standard myeloma regimens. Combinations with Rd or Pd in RRMM have demonstrated promising early results, with an ORR of 54% and 79% observed, respectively [64].

#### 3.1.3. B-Cell Maturation Antigen (BCMA)

B-cell maturation antigen (BCMA; TNFRSF17) is a member of the tumour necrosis factor receptor superfamily and plays a significant role during plasma cell differentiation and survival via interaction with its ligands, BAFF and APRIL [65,66]. BCMA is selectively expressed by plasmablasts and differentiated PCs but not by B lymphocytes, haematopoietic stem cells and non-haematopoietic tissue [67]. Overexpression of BCMA in MM murine models resulted in the upregulation of genes associated with osteoclast activation, adhesion, angiogenesis/metastasis and immunosuppression [65]. Additionally, compared to healthy donors, elevated levels of serum BCMA (sBCMA) have been identified in patients with plasma cell dyscrasias, where the levels correlated with the plasma cell burden in bone marrow biopsies, clinical course and changes in paraprotein [68]. sBCMA levels above the median were predictive of a significantly shorter PFS and OS in MM patients. These data have highlighted BCMA as an attractive target in myeloma; however, studies have also demonstrated that normal plasma cells can survive BCMA suppression, thought mainly via a compensatory mechanism of upregulation of its co-family receptor TACI [69]. Suppression of BCMA in malignant plasma cells has shown variable effects [70,71]. Whilst targeting BCMA with monoclonal antibodies has shown some efficacy in killing myeloma cells in vitro, a range of alternate methods of targeting BCMA are in clinical trials and include ADCs, bi-specific T-cell engagers (BiTE) and chimeric antigen–receptor T-cell (CAR-T) conjugates [72].

Belantamab mafodotin (GSK2857916) is the first therapeutic anti-BCMA ADC consisting of a humanised IgG1 anti-BCMA antibody conjugated with the toxin monomethyl auristatin F (MMAF) [73]. Upon binding to BCMA on the cell surface, belantamab mafodotin is rapidly internalised, and the cytotoxic MMAF is released, resulting in apoptosis [74]. Preclinical data also demonstrates that macrophage-mediated phagocytosis of MM cells is induced by the agent [69]. The first in-human phase I (DREAMM-1) study evaluated belantamab mafodotin in RRMM patients, of which 89% were double refractory to PIs and IMiDs, and 37% were refractory to daratumumab. A promising ORR of 60% was observed, with a median PFS of 12 months and a median duration of response of 14.3 months. Grade 1/2 corneal events were a frequent occurrence throughout the study, with the most common grade 3/4 events, thrombocytopenia and anaemia [75]. The follow-up phase II DREAMM-2 study examining different dosing regimens failed to replicate the response rates in DREAMM-1, with ORR of 32–34%. The main difference in participants characteristics was that almost all patients in DREAMM-2 had been exposed and were refractory to daratumumab [76]. The mechanism by which CD38 monoclonal exposure and resistance appear to lead to loss of effectivity of belantamab remains unclear.

A number of clinical trials utilising belantamab mafodotin in combination with other anti-myeloma agents are currently active or recruiting. The DREAMM-6 study is an open-label phase I/II study evaluating the safety, tolerability and efficacy of belantamab mafodotin when administered in combination with Rd or Vd in patients with RRMM who have become refractory or relapsed after one or more prior treatment lines [77]. Preliminary results from DREAMM-6 demonstrate that belantamab mafodotin in combination with Vd results in an overall response rate of 78%, with 50% of patients experiencing at least a very good partial response (VGPR). These results suggest that, like CD38 monoclonal antibodies, the combination of belantamab mafodotin with established therapies in myeloma can increase the response rates.

A second method of targeting BCMA is via BiTE antibodies which operate by forming an immunological synapse between a tumour antigen and a CD3+ T-cell, resulting in T cell binding to the tumour cell, activation and tumour lysis [78]. To date, at least eight BCMA BiTE antibodies are currently in clinical development, many of which are still recruiting in phase I dose escalation trials [79]. Recent phase I trials as single agents demonstrate that different BiTE antibodies have objective response rates between 70 and 83% at optimal dosing levels [80,81]. More data and longer follow-up are required, but the promising single-agent efficacy with acceptable toxicity profile has led to a number of proposals combining BCMA targeting BiTE antibodies in combination with standard therapies.

A third method of targeting BCMA has also been explored in MM and uses CAR-T cell therapy. CAR-T therapy involves the cultivation of T-cells harbouring a synthetic receptor directed against a tumour-associated antigen that is independent of the major histocompatibility complex [82]. CAR-T therapies directed against CD19 have shown promising success in refractory B-cell malignancies [83,84]. Over 20 different anti-BCMA CAR-T products have been developed and are undergoing clinical assessment. BCMA CAR-T cells have recently been discussed in a systematic review and meta-analysis, [85] with detailed review outside the scope of this article. In brief, this review assessed the safety and clinical efficacy of BCMA CAR-T therapy in 640 MM patients and revealed a pooled ORR of 80.5%, with a CR observed in 44.8% and a median PFS of 12.2 months. However, significant toxicity was observed, including cytokine release syndrome in 80.3% and neurotoxicity in 10.5% of the patients. Nevertheless, BCMA-targeted CAR-T therapy appears highly efficacious in advanced MM. A summary of recent studies presented at the American Society of Haematology Scientific Meeting in December 2020 can be seen in Table 1. Similar to the previously mentioned meta-analysis, these studies show very promising response rates and duration of response, albeit the numbers of patients treated remain small, with short duration of follow-up. We await with anticipation further later phase trials which are currently recruiting for BCMA CAR-T cells.

#### 3.1.4. SLAMF7

Signalling lymphocytic activation molecule (SLAM) family receptors are highly expressed in haematopoietic cells and serve an important role in the regulation of the normal immune system [95]. SLAMF7 is a cell surface glycoprotein receptor that is expressed on NK cells, CD8+ T cells, activated B-cells and dendritic cells, as well as normal and malignant PCs [96]. It has distinctive features not found in other members of the SLAM family that make it a compelling therapeutic target in myeloma. Additionally, increased expression of SLAMF7 is observed in MM cells independent of molecular profile, molecular subtype or the presence of cytogenetic abnormalities [96,97]. Its role in myelomagenesis is supported by SLAMF7 overexpression inducing increased MM cell adhesion to bone marrow stromal cells, cyclin D2-dependent proliferation and VEGF-induced bone marrow angiogenesis in preclinical models [98]. High levels of soluble SLAMF7 (sSLAMF7) have been correlated with more aggressive clinical characteristics and shorter PFS times when compared to sSLAMF7-negative MM patients. Furthermore, sSLAMF7 levels are undetectable or decreased following treatment, highlighting its role as a potential biomarker for MM therapy [99].

Elotuzumab is a humanised IgG1 monoclonal antibody that targets the extracellular domain of SLAMF7, with limited interaction with other members of the SLAM family. Elotuzumab induces selective lysis of myeloma cells via activation of NK cells. The Fab portion of elotuzumab binds SLAMF7 expressed on malignant PCs, while the Fc portion of the drug binds the activating Fc receptor, CD16, present on NK cells. This interaction triggers NK cell activation via recruitment of the adaptor protein EAT2 and the release of cytotoxic granules against the tagged tumour cells [100]. Due to the absence of EAT2 in MM cells, elotuzumab engagement does not result in activation of tumour cells. Additionally, elotuzumab reduces MM cell adhesion in the bone marrow microenvironment [97].

Elotuzumab as a single agent demonstrates a modest response, as shown in the initial phase I study in 35 RRMM patients, where 9 patients (26.5%) obtained stable disease, and the remaining experienced progressive disease (PD) [101]. Phase I combination studies of elotuzumab with either Vd or Rd have demonstrated greater synergistic efficacy, with ORR of 48% and 82%, respectively [102,103]. In the phase III ELOQUENT-2 study, elotuzumab was examined in combination with lenalidomide and dexamethasone (ERd versus Rd) in RRMM having previously received 1–3 lines of prior therapy [104]. The ORR in the elotuzumab group was 79%, while it was 66% in the control group (*p* < 0.001), with a median PFS of 19.4 versus 14.9 months in the elotuzumab group. The final overall survival results of ELOQUENT-2 demonstrated a statistically significant 8.7-month improvement in OS with ERd versus Rd (median 48.3 vs. 39.6 months, *p* = 0.0408) [105]. This improvement in OS was greatest amongst subgroups generally associated with poorer outcomes, including patients with IMWG high-risk disease (median, 29.8 vs. 24.8 months), disease refractory to the last prior therapy (median, 40.4 vs. 25.9 months), ISS stage III disease (median, 21.7 vs. 14.0 months) and adverse cytogenetic abnormalities. The phase II ELOQUENT-3 examining elotuzumab in addition to pomalidomide and dexamethasone (EPd versus Pd) demonstrated an ORR of 52% in the elotuzumab arm as compared with 26% in the control group. The median PFS was 10.3 months, versus 4.7 months in the elotuzumab group versus the control group [106]. Importantly, the addition of elotuzumab to lenalidomide or pomalidomide in the above studies was not associated with increased toxicity. The addition of elotuzumab to Vd in a phase II clinical trial showed a trend towards increased OS and PFS (median, 9.7 vs. 6.9 months, *p* = 0.09) but did not reach statistical significance [107].

The evaluation of elotuzumab combinations in untreated, newly diagnosed MM patients has yielded underwhelming results. Data from the phase III ELOQUENT-1 study evaluating ERd versus Rd in newly diagnosed, previously untreated and transplant-ineligible patients have not shown a statistically significant improvement in PFS, as reported by Bristol-Myer-Squibbs. A full evaluation of the ELOQUENT-1 data will be presented at a future meeting. A second study, SWOG-1211, is a randomised phase II trial comparing RVd with or without elotuzumab in patients with untreated, high-risk multiple myeloma. No difference in median PFS was observed between RVd–elotuzumab and RVd (31.47 vs. 33.64 months) [108]. Overall, elotuzumab appears most effective in relapsed/refractory patients in combination with an established regimen containing IMiDs, where it has shown minimal toxicity and improved survival rates.

### 3.2. Inhibitors of Nuclear Cytoplasmic Transport Receptors: Exportin 1

Exportin 1 (XPO1; CRM1) is a ubiquitous nuclear–cytoplasmic transport receptor that serves as a carrier protein enhancing transportation from the nucleus to the cytoplasm. Dysregulation of XPO1 in cancer results in the export from the nucleus of tumour suppressor proteins that have a critical growth inhibitory and apoptotic role, in turn sequestrating them within the cytoplasm and promoting a pro-tumour state [109]. Furthermore, overexpression of XPO1 correlates with a poor prognosis or resistance to chemotherapy in a range of solid tumour sand haematological malignancies. Increased XPO1 expression correlates with disease progression in MM, lytic lesions, short survival and PI resistance [110,111].

Selinexor (KPT-330) is an orally bioavailable, selective inhibitor of XPO1-mediated nuclear export. The phase II (STORM) trial examined selinexor in conjunction with oral dexamethasone in a heavily pre-treated population refractory to over four lines of prior therapy. The ORR was 21%, with a median PFS of 5 months and 65% of responding patients alive at 12 months [112]. Pre-clinical studies have demonstrated the synergistic antimyeloma activity of selinexor and proteasome inhibitors via suppression of NFkB signalling and the nuclear retention of tumour suppressor genes [113]. The phase II BOSTON study evaluating selinexor with low-dose bortezomib and dexamethasone (SVd) demonstrated an ORR of 63% with a median PFS of 9.0 months. Response rates were higher in proteasome inhibitor-non-refractory compared to proteasome inhibitor-refractory patients (84% vs. 43%, respectively). The regimen was well tolerated, with most adverse events being grade 1/2 [114]. Data from the follow-on phase III BOSTON study comparing the addition of selinexor to bortezomib and dexamethasone (SVd vs. Vd) were presented at the 2020 ASCO Virtual Program [115]. The study met its primary endpoint of a statistically significant increase in PFS with a median duration of 13.93 vs. 9.46 months in the SVd vs. Vd groups, respectively. This was true for all subgroups, including patients with 17p deletions and those previously treated with lenalidomide. Response rates were also significantly higher with SVd (76.4% vs. 62.3%, *p* = 0.0012). Median OS was not reached in the SVd group and was 25 months in the Vd arm. Peripheral neuropathy ≥ 2 was less with SVd (21% vs. 34%, *p* = 0.0013), but the triple combination was associated with higher thrombocytopenia and more fatigue, nausea and treatment-related discontinuations. The STOMP trial is a multi-arm study evaluating three-drug regimens involving selinexor in combination with IMiDs, PIs and daratumumab. Early data from the arm utilizing selinexor and weekly carfilzomib plus dexamethasone in carfilzomib-naïve patients with a median four lines of prior therapy yielded an ORR of 72%. With a median follow-up period of 4.7 (1.8–16.3) months, the median PFS has not been reached [116].

The combination of selinexor, pomalidomide and dexamethasone (SPd) demonstrated promising results, with an ORR of 58% in lenalidomide-treated or refractory and pomalidomide-naïve MM patients compared to 31% ORR with Pd in a similar population, as previously published [117]. A median PFS of 12.12 months was observed in the SPd arm in a population with a median four lines of prior therapy. All treatment-associated adverse events were expected and manageable with appropriate supportive care (e.g., G-CSF). Although the ORR is similar to that of previously published trials with pomalidomide-based triplet therapy, the ability to provide an oral triplet regimen is an attractive and advantageous option. Overall, early studies of selinexor suggest it is an efficacious therapy and shows promising results in combinations. Its novel method of action and parenteral mode of delivery makes it an encouraging addition to myeloma therapy.

### 3.3. BCL-2 Family Proteins

BCL-2 and its family members function as critical regulators of apoptosis and have been shown to play critical roles in myeloma survival [118,119,120,121]. Venetoclax (ABT-199) is a selective, orally bioavailable BCL-2 inhibitor with an expanding role in the treatment of both lymphoid and myeloid malignancies [122]. A subset of MM patients with t(11;14) translocations demonstrate a high dependency on BCL-2, and deeper, prolonged responses are observed in this subgroup. In a phase I study of venetoclax monotherapy in 66 patients with RRMM, the ORR was 21% (14/55), and 15% of the patients achieved a VGPR or better [123]. The majority of responses (12/14 [86%]) were reported in patients within the t(11;14) subgroup. Within this group, the ORR was 40%, with 26% of the patients achieving a VGPR or better. Further clinical evaluation in t(11;14)-positive RRMM patients demonstrated an ORR of 46%, and >VGPR was observed in 26% of the patients [124]. The nine-month estimates for PFS and OS were 57% and 71%, respectively. Consequently, this combination is being investigated in the ongoing phase III CANOVA trial in t(11;14)-positive RRMM [125].

Venetoclax in combination with other agents has also shown efficacy in t(11;14)-negative patients. Upregulation of MCL-1 confers venetoclax resistance, which is abrogated by the addition of bortezomib in pre-clinical myeloma models [126]. The phase III BELLINI study assesses the efficacy of venetoclax or placebo in combination with Vd in 291 patients with RRMM [127]. With a median follow-up of 18.7 months, median PFS was greater with venetoclax (22.4 versus 11.5 months; *p* = 0.010). Median OS was not reached in either group. Increased mortality was observed in the venetoclax group, predominantly due to increased rates of infection. The unexpected finding of increased mortality in the venetoclax group has complicated the use of venetoclax in combination with other therapies in myeloma; more in-depth analysis and further measures to minimise infection risk are required in the future. Given these findings, the role of BCL-2 inhibition remains unclear within the myeloma field.

### 3.4. HDAC Inhibitors

**Figure 1:**
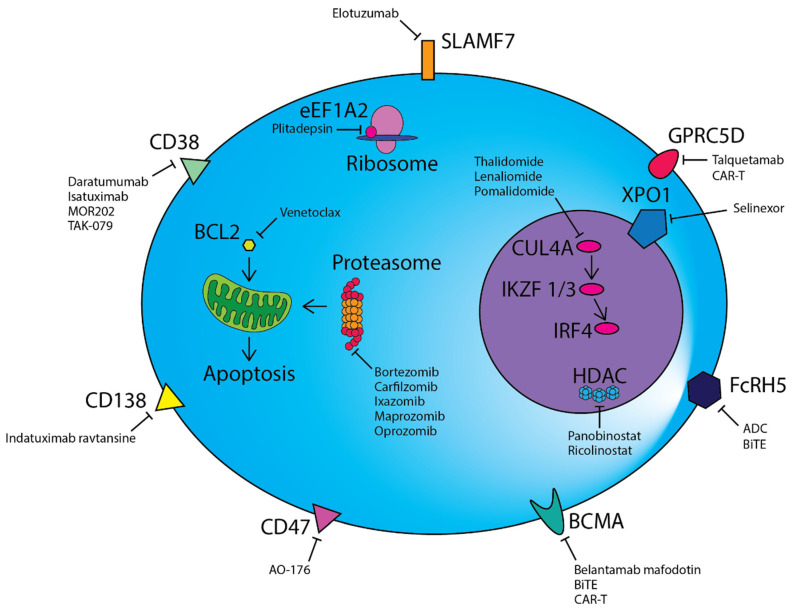
Therapeutic modalities in multiple myeloma.

HDACs are critical epigenetic regulators of gene expression that function through the modification of histones. Panobinostat, a pan-HDAC inhibitor, has been approved by the FDA as a third-line treatment for RRMM patients previously treated with PI and IMiD. Panobinostat in conjunction with intravenous bortezomib and dexamethasone was examined in a phase III clinical trial (PANORAMA-1) and demonstrated efficacy in RRMM, with an improvement in median PFS and OS of four months [128]. However, serious adverse events were reported in 60% of patients in the panobinostat group compared to 42% in the placebo group. PANORAMA-3, a phase II study examining three different dosing regimens of panobinostat with subcutaneous (SC) bortezomib and dexamethasone, has demonstrated a more favourable safety profile, suggesting that SC bortezomib improves the tolerability of the regimen [129]. The greatest ORR was observed in the three-time weekly group (62.2%), at the expense of increased adverse events. Increased interest has been aimed at more selective HDAC inhibitors due to the side effects observed that often lead to treatment discontinuation. The newer selective inhibitor of HDAC6, ricolinostat (ACY-1215), appears to be one of the more promising agents in RRMM. The single agent ricolinostat in combination with bortezomib and dexamethasone in phase I/II trials showed an ORR of 37% [130]. In a multicentre phase Ib study in combination with lenalidomide and dexamethasone, the overall response rate was 55% [131]. Both studies reported favourable tolerability and limited related adverse events. Figure 1 shows the therapeutic targets in myeloma.

CD38, CD47, CD138, SLAMF7, GPRC5D, FcRH5 and BCMA are highly expressed on the surface of malignant plasma cells and are targeted by a range of different monoclonal antibodies or antibody–drug conjugates. Chimeric antigen receptor T-cell (CAR-T) and bispecific T-cell engager (BiTE) cellular therapies directed against BCMA, GPRC5D and FcRH5 are also a novel therapeutic avenue. Proteasome inhibitors prevent the degradation of misfolded proteins via the proteasome, resulting in eventual cell death due to the unfolded protein response. BCL2 inhibitors such as venetoclax induce mitochondrial outer membrane permeabilization, culminating in apoptosis. IMiDs alter the target specificity of Cereblon (CRBN) as part of an E3 ubiquitin ligase complex and lead to the degradation of Ikaros (IKZF1) and Aiolos (IKZF3), which consequently results in a reduction in IRF4, a key mediator of the oncogene MYC. Plitadepsin binds to eEF1A2 in the ribosome and abrogates its pro-oncogenic moonlighting effects. Additional therapies directed against targets within the nucleus include the nuclear transport protein XPO-1 and epigenetic regulators such as histone deacetylases (HDACs).

### 3.5. Plitadepsin

Plitadepsin (Aplidin^®^) is a synthetically produced cyclic depsipeptide that exerts its anticancer effects by binding to eukaryotic Elongation Factor 1A2 (eEF1A2), a protein which is overexpressed in a range of tumours, including MM [132]. The predominant function of eEF1A2 involves the delivery of aminoacyl-tRNAs to the ribosome, but it also demonstrates pro-oncogenic moonlighting effects including inhibition of apoptosis, protein degradation by the proteasome, heat shock response and regulation of oxidative stress [132,133]. Plitadepsin also binds to the Rac1 GTPase, resulting in the sustained activation of c-Jun-N-terminal kinase (JNK) and p38 mitogen-activated protein kinases (p38/MAPK) signalling pathways, culminating in caspase-dependent apoptosis [134].

Plitadepsin was administered intravenously alone or in combination with dexamethasone in a phase II study evaluating its clinical and safety profile in RRMM [135]. In 47 of 51 patients evaluable for efficacy, the ORR was 13% with plitadepsin alone, while it was and 22% in the cohort of patients also receiving dexamethasone (*n* = 19). Plitadepsin was well tolerated both alone or in combination, with anaemia (29%) and thrombocytopenia (18%) being the most frequent grade 3/4 haematologic toxicities. Non-haematological adverse events included fatigue (16%), muscular toxicity (6%), transient AST/ALT elevation (27%) and CK increases (23%). Following on from these results, the randomized phase III ADMYRE trial evaluated plitadepsin plus dexamethasone alone in RRMM after at least three prior therapeutic regimens [136]. Median PFS was 2.6 months in the plitadepsin group and 1.7 months in the control arm (*p* = 0.0054), with a median OS of 11.6 versus 8.6 months (*p* = 0.1261). OS improvement favouring plitadepsin was observed when discounting the crossover effect (37 patients crossed over from the control group) (*p* = 0.0015). The most common grade 3/4 toxicities were fatigue (10.8%), myalgia (5.4%) and nausea (3.6%) in the plitadepsin arm.

A phase I trial combining plitadepsin with Vd in 18 evaluable patients with RRMM produced an ORR of 56%, with 33% of the patients achieving at least a VGPR [137]. A median PFS of 8.3 months was observed. No dose-limiting toxicities were observed, and grade 3/4 haematological toxicities included anaemia (25%), neutropenia (25%) and thrombocytopenia (60%). Non-haematological toxicities were mild.

Although single-agent efficacy is underwhelming, the safety profile of plitadepsin does not appear to present the same toxicities associated with other anti-myeloma agents. Additionally, the novel mechanism of action may prove that plitadepsin in combination with other anti-myeloma agents may be an alternative option for RRMM.

### 3.6. Other Novel Targeted Therapies

CD47 is an immune checkpoint that is overexpressed in a number of haematological and solid tumours. Macrophages express the checkpoint receptor, signal regulatory protein α (SIRPα/CD172a), which recognizes CD47 on target cells. Interactions between CD47 and SIRPα generate a “don’t eat me” signal by initiating a signalling cascade that eventuates with the inhibition of macrophage phagocytic activity [138]. CD47 expression is markedly higher in MM cells compared to other constituents of the bone marrow milieu, and its levels appear to correlate with disease progression from normal PCs to MM [139]. In vitro inhibition of CD47 using an anti-CD47 antibody (Vx1000R) induced phagocytosis as early as 4 h after treatment, which continued to increase over 24 h [139]. AO-176 is a novel anti-CD47 antibody that exerts substantial single-agent in vivo anti-tumour activity in MM xenograft models [140]. A phase I study of AO-176 in MM is currently recruiting (NCT04445701). Overall, these results highlight targeting CD47 as a novel and promising strategy in the treatment of MM. However, we cautiously await further validation from clinical trials, given the previous experience of checkpoint blockade of PD-1 in MM, with pre-clinical data not translating to clinical efficacy and the appearance of significant adverse events [141,142].

The orphan G protein-coupled receptor, class C group 5 member D (GPRC5D) is expressed on malignant PCs, whereas its expression in normal tissue is limited to the hair follicle [143]. In preclinical models, GPRC5D-targeted CAR-T therapy demonstrated in vivo activity in MM xenograft mice, with comparable tumour regression compared to BCMA-targeted CAR-T therapy [143]. Phase I evaluation of a first-in-class bispecific antibody that binds to GPRC5D and CD3 (talquetamab) in RRMM was recently presented at the ASH 2020 meeting [144]. ORR for IV doses was 78% (14/18), whilst SC administration demonstrated an ORR of 67% (8/12). Responses were durable, and the median was not reached in 36/46 patients. Four patients demonstrated a response lasting over 15 months, with the current longest response being over 23 months. The most common grade 3/4 AEs were lymphopenia (37%), anaemia (27%) and neutropenia (25%). Cytokine release syndrome (CRS) was predominantly grade 1/2, except for five patients with grade CRS who received IV dosing. A maximum tolerated dose has not been defined. The encouraging pre-clinical and clinical activity of GPRC5D CAR-T or BiTE therapies support ongoing monotherapy development and combination approaches.

Fc receptor-homolog 5 (FcRH5; also known as FcRL5, IFGP5, CD307 and IRTA2) is a cell surface antigen of unknown function whose expression is restricted to mature B cells, with greater expression on malignant PCs [145,146]. DFRF4539A is an ADC that contains a humanized IgG1 anti-human FcRH5 monoclonal antibody (MFRF3266A) and a potent anti-mitotic agent, monomethyl auristatin E. A phase I study of DFRF4539A in RRMM showed limited clinical activity with 34 of 37 patients demonstrating SD or PD [147]. Consequently, further investigation of DFRF4539A has ceased. An FcRH5 BiTE antibody (BFCR4350A) has demonstrated promising results in primates, with complete depletion of BM PCs without severe or prolonged cytokine release [148]. A phase I study assessing BFCR4350A is currently recruiting (NCT03275103).

## 4. Conclusions

Over the last decade, a plethora of novel agents have become available for the treatment of myeloma (Figure 1). With the potential exception of CAR-T cells, they all have demonstrated some, but limited, efficacy as single agents in RRMM. As a result, many have been trialled and shown to be efficacious either when combined with established myeloma therapies, in specific subgroups of patients, or in earlier stages of the disease. Excitingly, many of these new agents have unique modes of action, and most have acceptable toxicity profiles, with relative specificity for targeting myeloma cells. Despite the therapeutic options these agents provide, there are a number of questions which remain unanswered. Firstly, determining which of these agents, in conjunction with established therapies, will be the ideal treatment for myeloma patients by balancing optimal efficacy with minimal toxicity; secondly, in which order should these therapies be used in patients with myeloma to achieve the best responses and survival outcomes; lastly, if the potential for cure for myeloma lies within the combination of therapies discussed within this article. Answering these three major questions, as well as the ongoing discovery of new therapies, are the major challenges of the future for myeloma clinicians, researchers and patients alike.

## Figures and Tables

**Table 1:** CAR-T cell studies presented as oral presentations at the American Society of Haematology Scientific Meeting December 2020.

Authors	Product	Total Number of Patients *	ORR of All Patients	Duration of Response	Optimal Cell Dose	Number of Patients with Optimal Dose	ORR at Optimal Dose	Median Duration of Follow Up
Mailankody et al. [86]	Allogeneic BCMA CAR-T cells with CD52 monoclonal antibody	27	11/27 (40%)	Not reported	3.2 × 10^8^	10	6/10 (60%)	3.2 months
Alsina et al. [87]	Autologous BCMA CAR-T cells culture with PI3K inhitibor	59	40/59 (68%)	17 months (estimated)	4.5 × 10^8^	33	24/33 (73%)	5.8 months
Lin et al. [88]	Autologous BCMA CAR-T cells	62	47/62 (75.8%)	10.3 months	8 × 10^8^	3	3/3 (100%)	18.1 months
Hao et al. [89]	Autologous fully humanised BCMA CAR-T cells	24	21/24 (87.5%)	21.8 months	1.5 × 10^8^	21	Not reported	17.7 months
Kumar et al. [90]	Autologous BCMA CAR-T cells	18	17/18 (94%)	Not reported	2.5–3 × 10^8^	6	Not reported	6 months
Costello et al. [91]	Autologous BCMA CAR-T cells manufactured with nanoplasmids	30	17/30 (57%)	Not reported	8.47 × 10^8^	4	3/4 (75%)	Not reported
Madduri et al. [92]	Autologous BCMA CAR-T cells	97	94/97 (96.9%)	Not reached	0.5–1.0 × 10^6^/kg	97	94/97 (96.9%)	12.4 months
Jiang et al. [93]	Autologous BCMA/CD19 dual CAR-T cells	16	15/16 (93.6%)	Not reported	3 x 10^5^/kg	6	6/6 (100%)	7.3 months
An et al. [94]	Second-generation Autologous BCMA CAR-T cells	23	22/23 (95.7%)	Not reached	4.5–6 × 10^6^/kg	9	8/9 (88.9%)	6.2 months

ORR = overall response rate, * total number of patients refers to total number of evaluable patients, optimal cell dose was determined by the investigators.

## Data Availability

Not applicable.
